# PaCO_2_: a treatable trait in acute respiratory failure

**DOI:** 10.3389/fmed.2026.1773798

**Published:** 2026-05-11

**Authors:** Carmelo Dueñas-Castell, José Correa-Guerrero, Dairo Rodelo-Barrios, Elber Osorio-Rodríguez, Miguel Aguilar-Schotborgh, Amilkar Almanza-Hurtado, Diana Borré-Naranjo, Guillermo Ortiz-Ruiz

**Affiliations:** 1 Faculty of Medicine, Universidad Metropolitana, Barranquilla, Colombia; 2 Department of Critical Medicine and Intensive Care, University of Cartagena, Cartagena de Indias, Colombia; 3 Department of Critical Medicine and Intensive Care, Clínica Gestión Salud, Cartagena de Indias, Colombia; 4 Department of Critical Medicine and Intensive Care, Hospital Universitario del Caribe, Cartagena de Indias, Colombia; 5 Department of Critical Medicine and Intensive Care, Faculty of Medicine, Simón Bolívar University, Barranquilla, Colombia; 6 Critical and Internal Medicine Research Group (CRITIMED), Cartagena de Indias, Colombia; 7 Department of Intensive Medicine, Clínica Iberoamérica, Barranquilla, Colombia; 8 Group Care Medicine, Clínica Colsanitas, Bogotá, Colombia; 9 Department of Pneumology, University of Javeriana, Bogotá, Colombia; 10 Intensive Care and Obstetrics Research Group (GRICIO), University of Cartagena, Cartagena de Indias, Colombia; 11 Department of Internal Medicine and Critical Medicine, Hospital Serena del Mar, Cartagena de Indias, Colombia; 12 Department of Critical Medicine and Intensive Care, University of Bosque, Bogotá, Colombia

**Keywords:** carbon dioxide, hypercapnia, hypocapnia, noninvasive ventilation, respiratory insufficiency

## Abstract

**Introduction:**

Treatable traits are a cornerstone of personalized (precision) medicine. Arterial carbon dioxide tension (PaCO_2_) is a fundamental measure in acute respiratory failure. We aimed to determine whether PaCO_2_ can be considered a treatable trait in patients with acute respiratory failure.

**Methods:**

We performed an electronic search to identify studies addressing hypercapnia and hypocapnia as clinically actionable features in respiratory insufficiency, including their prognostic value and role as potential treatment targets. A systematic snowball approach (backward and forward citation tracking) was applied to capture additional relevant articles. Evidence was synthesized narratively to contextualize findings within the conceptual framework of PaCO_2_ as a treatable trait.

**Results:**

PaCO_2_ supports classification of acute respiratory failure as hypercapnic or non-hypercapnic, informs prognosis, and helps define non-invasive management with high-flow nasal cannula or non-invasive ventilation. In intubated patients, PaCO_2_ contributes to monitoring ventilatory strategies such as lung-protective ventilation and prone positioning, and it helps interpret outcome-relevant variables such as ventilatory ratio and mechanical power. Across the peri-extubation period, PaCO_2_ is associated with clinically meaningful outcomes before, during, and after ventilator weaning and can guide selection of post-extubation non-invasive support to reduce reintubation risk.

**Discussion:**

PaCO_2_ may be considered a treatable trait across the pre-intubation, invasively ventilated, and post-extubation phases of acute respiratory failure. Further research is needed to define clinically actionable limits and targets in relevant patient subgroups, including safety margins in relation to pH and exposure time.

## 1 Introduction

The “Oslerian paradigm” frames clinical diagnosis as the recognition of a syndrome or disease from bedside findings, supported by anatomical, physiological, or histopathological data (1). However, the marked heterogeneity of syndromes such as sepsis and acute respiratory distress syndrome (ARDS), together with the large number of failed pharmacological and non-pharmacological interventions in critically ill patients, has accelerated the shift toward personalized (precision) medicine (2).

Precision medicine has been defined as “treatments directed to the needs of each patient based on distinctive genetic, phenotypic, psychosocial characteristics or biomarkers that differentiate them from other individuals with similar clinical pictures” (3, 4). Within this framework, approaches such as *treatable traits* have been proposed to guide therapy. This concept appears to have been introduced by Dr. Claus Vogelmeier (University Hospital of Marburg) to “facilitate an even more personalized management of the patient,” proposing that treatment could be organized around a trait rather than a disease label (5). In chronic obstructive pulmonary disease (COPD), he highlighted three inflammatory markers—neutrophils, eosinophils, and C-reactive protein—as potential treatable traits. Neutrophilia could be addressed through smoking cessation or azithromycin; eosinophilia may respond to corticosteroids; and elevated C-reactive protein could be targeted with statins (5). Notably, given the clinical overlap between asthma and COPD, identifying such treatable traits may be as relevant—or even more relevant—than assigning a diagnostic label when selecting therapeutic interventions (5, 6). A treatable trait has four key characteristics (6, 7):

1. Clinical relevance, with an association to meaningful outcomes (e.g., symptoms, health status, or risk of future events).
2. Identifiability and measurability, using practical and reproducible assessments.
3. Treatability, with an available intervention that can modify the trait.
4. Responsiveness to intervention, with evidence that modifying the trait leads to clinically meaningful improvement in patient outcomes.

**Figure 1:**
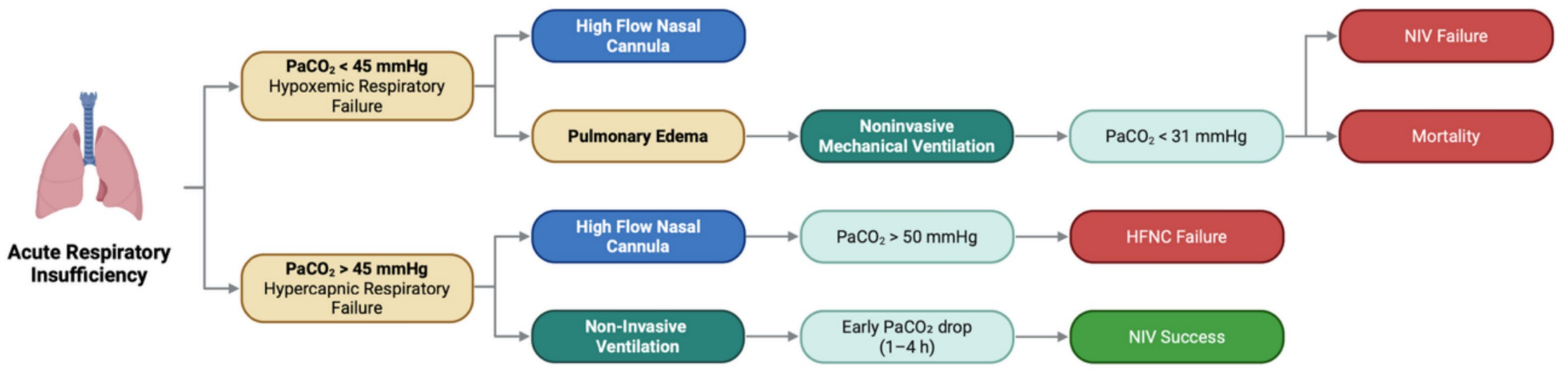
PaCO_2_ for the diagnosis and decision-making regarding non-invasive support of acute respiratory failure. Created by the authors.

Within this framework, arterial carbon dioxide tension (PaCO_2_) may be considered a potential treatable trait in acute respiratory failure. The respiratory system is fundamentally responsible for gas exchange, ensuring oxygen uptake and carbon dioxide (CO_2_) elimination (8). When this function becomes impaired, acute respiratory failure (ARF) ensues (8–10). Clinically, ARF is often described according to the predominant gas-exchange abnormality, although hypoxemia and hypercapnia may coexist in mixed presentations. In this context, PaCO_2_ remains a central physiological variable: it is often low or normal in predominantly hypoxemic forms of ARF, whereas it is elevated in hypercapnic presentations. However, PaCO_2_ should be interpreted together with pH, oxygenation, and the overall clinical setting, since an elevated PaCO_2_ may reflect acute, chronic, or compensated disturbances rather than acute hypercapnic respiratory failure alone. This distinction is clinically important because it helps define the underlying physiological profile and supports therapeutic decision-making (10) (Figure 1).

## 2 Methods

An initial electronic search was conducted in PubMed/MEDLINE, Scopus, and Web of Science using the following terms: (“Carbon Dioxide” [MeSH] OR PaCO_2_ OR “partial pressure of carbon dioxide” OR hypercapnia OR capnography OR “end-tidal CO_2_” OR ETCO_2_ OR VCO_2_) AND (“Respiratory Insufficiency” [MeSH] OR “respiratory failure” OR “acute respiratory failure” OR “acute hypercapnic” OR “COPD exacerbation” OR “acute exacerbation of COPD” OR ARDS OR “acute respiratory distress syndrome” OR “critical illness” OR ICU OR “intensive care”) AND (phenotype OR “treatable trait” OR “treatable traits” OR biomarker OR “treatment target” OR “prognostic factor” OR “predictive factor”). This strategy identified key articles.

From these key studies, a snowball approach was systematically applied in both directions. For backward searching, the reference lists of each key study were screened to identify additional relevant articles. For forward searching, we screened articles that cited the key studies. Studies were then grouped according to study design, study objective, definitions of hypercapnia and hypocapnia, and outcomes. Findings are presented as a narrative synthesis to contextualize the evidence within the conceptual framework of CO_2_ as a treatable trait. For clarity, throughout this manuscript, “non-invasive respiratory support” is used as a general term that may include high-flow nasal cannula (HFNC), continuous positive airway pressure (CPAP), and non-invasive ventilation with pressure support. Whenever the available evidence predominantly pertains to one specific modality, that modality is named separately.

## 3 Results

Table 1 summarizes the studies evaluating PaCO_2_ abnormalities across common acute care settings, including invasive mechanical ventilation (ARDS/COVID-19), non-invasive ventilation (COPD/OHS and hypoxemic respiratory failure), post-extubation support, acute brain injury, and cardiogenic conditions. Across studies, definitions of dyscapnia varied, but recurrent thresholds included hypocapnia at PaCO_2_ < 35 mmHg (or ≤32-33 mmHg in some cohorts) and hypercapnia at PaCO_2_ > 45 mmHg, with higher cut-offs also reported (e.g., >50 mmHg or ≥54 mmHg during spontaneous breathing trials). Several non-invasive ventilation (NIV) studies incorporated respiratory acidosis using pH thresholds (typically pH < 7.35 or 7.25–7.35).

**Table 1:** Clinical studies on CO_2_ and their associated outcomes.

Reference	Type of study (*n*: number of patients)	Study objective	Definition of hypercapnia and/or hypocapnia	Outcomes
Kato et al. (19)	Retrospective observational study (*n* = 435)	To assess the relationship between PaCO_2_ and long-term mortality in patients with AHF	PaCO_2_ as a continuous variable and compared to cut-off points (31 mmHg and 40 mmHg)	PaCO_2_ < 31 mmHg was associated with higher risk of death (HR 1.71, 95% CI 1.05–2.79; *p* = 0.032).
				Long-term mortality: 26.4% (115/435).
Carrillo-Aleman et al. (20)	Retrospective observational study (*n* = 1,138)	To analyze whether hypocapnia is a risk factor for non-invasive ventilation (NIV) failure and in-hospital mortality in patients with cardiogenic pulmonary oedema (CPD)	Patients classified according to PaCO_2_: hypocapnic, eucapnic and hypercapnic (the exact value of hypercapnia is not specified in the abstract)	NIV failure: 15.4% (hypocapnic) vs. 10.7% (hypercapnic).
				In-hospital mortality: 18.7% (hypocapnic) higher than other groups.
Xu et al. (22)	Retrospective observational study (*n* = 1,029)	To explore the association between PaCO_2_ and NIV failure in patients with hypoxemic respiratory failure	Hypocapnia: PaCO_2_ ≤ 32 mmHg	NIV failure: 48% (PaCO_2_ ≤ 32) vs. 42% (PaCO_2_ > 32). Adjusted risk of NIV failure higher with hypocapnia (adjusted HR 1.23, 95% CI 1.01–1.49).
Díaz et al. (34)	Prospective, open-label, uncontrolled observational study (*n* = 681)	Evaluate NIV by reversing hypercapnic coma	Hypercapnia: PaCO_2_ > 45 mmHg	NIV success was 80%
González et al. (37)	Prospective observational study (*n* = 53)	To evaluate NIV response in hypercapnic and hypoxemic patients with COPD, OHSS, AHF	pH > 7.25 and PaCO_2_ > 45 mmHg	At 3 h of NIV: pH increased and PaCO_2_ decreased (*p* = 0.002; *p* = 0.03, as reported).
Rabec et al. (39)	Prospective observational study (*n* = 41)	To evaluate NIV in patients with sleep apnea and respiratory acidosis	pH < 7.35 and PaCO_2_ > 45 mmHg	Intubation was avoided in 95% of patients
Carrillo et al. (43)	Prospective observational study (*n* = 716)	To compare the efficacy of NIV in episodes of respiratory failure caused by OHSS and COPD.	pH < 7.35 and PaCO_2_ > 45 mmHg	NIV success: 88.4%
Li et al. (45)	Randomized controlled clinical trial (*N* = 320)	Efficacy of HFNC in COPD	pH > 7.35 and PaCO_2_ > 45 mmHg	19% of patients progressed to IMV
Tan et al. (46)	Randomized controlled clinical trial (*n* = 225)	Efficacy of HFNC/NIV in COPD Patients	pH 7.25–7.35 and PaCO_2_ > 50 mmHg	Progression to IMV: 25.7% (HFNC) vs. 14.3% (NIV).
Plant et al. (48)	Multicenter randomized controlled clinical trial (*N* = 236)	To assess whether NIV reduces intubation in acute exacerbation of COPD	pH 7.25–7.35 and PaCO_2_ > 45 mmHg	15% of patients progressed to IMV
Sellares et al. (49)	Randomized controlled clinical trial (*N* = 120)	Efficacy of NIV prolongation after resolution of respiratory failure	pH < 7.35 and PaCO_2_ > 45 mmHg	Respiratory failure recurred in 13%
Çiftci et al. (50)	Prospective observational study (*n* = 106)	To assess the feasibility of pressure support with assured average volume in patients with hypercapnic respiratory failure	pH < 7.35 and PaCO_2_ > 45 mmHg	NIV success: 76.4%
Nin et al. (54)	Prospective observational study (*n* = 889)	To evaluate the relationship between hypercapnia and mortality in patients with ARDS in the first 48 h	Hypercapnia: PaCO_2_ > 40 mmHg	aCO_2_ > 50 mmHg correlated with higher mortality (first 48 h).
Marik and Desai (38)	Observational, retrospective study (*n* = 61)	To evaluate BIPAP in patients with OHSS and BMI > 40 kg/m^2^	Hypercapnia: PaCO_2_ > 45 mmHg	37.7% progressed to IMV.
Duarte et al. (40)	Observational, retrospective study (*n* = 50)	To evaluate the outcomes of morbidly obese patients with respiratory failure treated with NIV and BMI > 35 kg/m^2^	Hypercapnia: PaCO_2_ > 50 mmHg	NIV success was 64%
Bry et al. (41)	Observational, retrospective study (*n* = 53)	To analyze the characteristics of subjects receiving long-term NIV after initial hospitalization for respiratory failure and BMI > 30 kg/m^2^	Hypercapnia: PaCO_2_ > 45 mmHg	NIV success was 90%
Piper and Sullivan (42)	Observational, retrospective study (*n* = 13)	To evaluate nocturnal nasal ventilation in obese patients with hypercapnia and BMI > 35 kg/m^2^	Hypercapnia: PaCO_2_ > 45 mmHg	NIV success was 64 69%.
Tiruvoipati et al. (53)	Retrospective, multicenter observational study (*n* = 252,812)	To assess the impact of hypercapnia in patients receiving IMV	Hypercapnia: PaCO_2_ > 45 mmHg	A PaCO_2_ > 45 mmHg was associated with higher mortality
Madotto et al. (63)	Prospective observational study (*N* = 2,813)	To determine the variability of CO2 in early ARDS, and its impact on outcomes.	Hypercapnia: PaCO_2_ > 45 mmHg	Mortality due to hypercapnia was 36%
			Hypocapnia: PaCO_2_ < 35 mmHg	Mortality due to hypocapnia was 38.1%.
Rusnak et al. (64)	Prospective observational study (*N* = 238)	To determine the effect of PaCO_2_ on 30-day all-cause mortality.	Hypercapnia: PaCO_2_ > 48.13 mmHg	Hypercapnia was not associated with increased mortality
			Hypocapnia: PaCO_2_ < 33 mmHg	Hypocapnia was associated with higher mortality.
Robba et al. (71)	Prospective observational study (*N* = 1,476)	To determine the effect of PaCO_2_ in patients with acute brain injury	Hypercapnia: PaCO_2_ > 45 mmHg	PaCO_2_ > 45 mmHg was associated with higher mortality.
			Hypocapnia: PaCO_2_ < 35 mmHg	PaCO_2_ < 35 mmHg was significantly associated with higher mortality.
Sellares J, et al. (83)	Prospective observational study (*N* = 181)	To determine whether PaCO_2_ is associated with prolonged weaning.	Hypercapnia: PaCO_2_ > 45 mmHg	Both cut-off points were associated with prolonged ventilatory weaning.
			Hypercapnia: PaCO_2_ > 54 mmHg	
Ferrer et al. (85)	Prospective observational study (*N* = 162)	To determine the relationship between hypercapnia and prolonged ventilatory weaning	Hypercapnia: PaCO_2_ > 45 mmHg	Higher rate of orotracheal re-intubation
Thille et al. (86)	Prospective observational study	To assess the rate of re-intubation in patients with HFNC or NIV in hypercapnic patients	Hypercapnia: PaCO_2_ > 45 mmHg	Lower rate of orotracheal re-intubation
Nava et al. (87)	Multicenter randomized controlled clinical trial (*N* = 122)	To assess the rate of re-intubation in patients at risk of developing post-extubation AKI with NIV for at least 8 h per day during the first 48 h in hypercapnic patients	Hypercapnia: PaCO_2_ > 45 mmHg	NIV was effective in preventing ventilatory failure after extubation.
Ferrer et al. (88)	Randomized clinical trial (*N* = 164)	To determine whether NIV prevents ventilatory failure and improves survival versus standard oxygen in hypercapnic patients	Hypercapnia: PaCO_2_ > 45 mmHg	NIV decreases the presence of acute ventilatory failure and mortality at 90 days after extubation compared to standard oxygen.
Girault et al. (89)	Randomized clinical trial (*N* = 388)	To assess the impact of NIV as a bridge to withdrawal of invasive ventilation in hypercapnic patients	Hypercapnia: PaCO_2_ > 45 mmHg or an increase in PaCO_2_ > 10% compared to the pre-extubation value	NIV decreased orotracheal re-intubation rate
Hilbert et al. (90)	Observational case–control study (*N* = 30)	To assess the efficacy of NIV in decreasing the rate of re-intubation and mortality in people with COPD.	Hypercapnia: 20% increase in measured baseline value after extubation and pH < 7.35	NIV decreased the rate of orotracheal re-intubation.
Fuller et al. (95)	Prospective observational study (*n* = 1,491)	To determine the association between PaCO_2_ and mortality during mechanical ventilation in the first 48 h	Hypocapnia (PaCO_2_ < 35 mmHg	Hypercapnia had a higher survival rate (85%) compared to hypocapnia (66%)
			Hypercapnia (PaCO_2_ > 45 mmHg)	
Braunsteiner et al. (62)	Observational, retrospective (*N* = 435)	Association between mechanical power, dyscapnia and mortality	Hypercapnia: PaCO_2_ > 50 mmHg	PaCO_2_ > 50 mmHg was not associated with increased mortality.
			Hypocapnia: PaCO_2_ < 35 mmHg	PaCO_2_ < 35 mmHg was associated with higher mortality
Tiruvoipati et al. (53)	Retrospective, multicenter observational study (*n* = 252,812)	To evaluate the impact of compensated hypercapnia and hypercapnic acidosis in patients receiving IMV	Hypercapnia (PaCO_2_ > 45 mmHg)	Hypercapnic acidosis (PaCO_2_ > 45 mmHg; pH < 7.35) within the first 24 h was associated with higher mortality
Nin et al. (54)	Prospective, non-interventional cohort study (*n* = 889)	To evaluate the relationship between hypercapnia and outcomes in mechanically ventilated patients with ARDS of <48 h’ duration	Hypercapnia (PaCO_2_ > 40 mmHg)	A PaCO_2_ > 50 mmHg was associated with higher mortality within the first 48 h.
Tsonas et al. (55)	Retrospective, multicenter observational study (*n* = 824)	To determine the incidence of hypercapnia and its associations with outcomes in mechanically ventilated patients with COVID-19	Hypercapnia (PaCO_2_ > 45 mmHg)	Hypercapnia (PaCO_2_ > 45 mmHg) was associated with a longer duration of mechanical ventilation and longer ICU and hospital length of stay.
Villar et al. (96)	Prospective cohort study (*n* = 253)	To evaluate early prediction of mechanical ventilation duration >14 days in patients with moderate-to-severe ARDS.	Hypercapnia (PaCO_2_ > 45 mmHg)	Changes in PaCO_2_ during the first 3 days of mechanical ventilation predict a duration of mechanical ventilation >14 days.
Madotto et al. (63)	Multicenter observational study (*n* = 2,813)	To determine the prevalence and impact of hypocapnia and hypercapnia on management and outcomes in patients with early ARDS.	Hypocapnia: PaCO_2_ < 35 mmHg	ICU mortality was higher with sustained hypocapnia in patients with mild-to-moderate ARDS.
			Hypercapnia: PaCO_2_ > 45 mmHg	
Rusnak et al. (64)	Prospective cohort study (*n* = 238)	To evaluate the prognostic impact of PaCO_2_ and PaO_2_ on 30-day all-cause mortality in patients with cardiogenic shock.	Hypocapnia: PaCO_2_ ≤ 33 mmHg	Hypocapnia (PaCO_2_ ≤ 33 mmHg) was associated with a higher risk of 30-day all-cause mortality.
Robba et al. (71)	Multicenter prospective observational study (*n* = 1,476)	To describe PaCO_2_ targets in patients with acute brain injury (ABI) and to assess the incidence of abnormal PaCO_2_ values during the first week of ICU stay.	Normocapnia: PaCO_2_ 35–45 mmHg	Severe hypocapnia (PaCO_2_ 26–31 mmHg) and hypercapnia (PaCO_2_ > 45 mmHg) were associated with a higher likelihood of in-hospital mortality.
			Mild hypocapnia: PaCO_2_ 32–35 mmHg	
			Severe hypocapnia: PaCO_2_ 26–31 mmHg	
			Forced hypocapnia: PaCO_2_ < 26 mmHg	
			Hypercapnia: PaCO_2_ > 45 mmHg	
Gattinoni et al. (73)	Retrospective analysis of a randomized controlled trial (*N* = 225)	To determine whether improvement in gas exchange in response to prone positioning is associated with better outcomes in acute lung injury (ALI) and ARDS.	Patients whose PaCO_2_ decreased by ≥1 mmHg after 6 h in the prone position.	Prone responders had a higher 28-day survival rate.
Pu et al. (82)	Multicenter prospective cohort study (*n* = 343)	To examine weaning from mechanical ventilation in ICU patients.	Hypercapnia: PaCO_2_ > 50 mmHg	Hypercapnia during the first spontaneous breathing trial was associated with prolonged mechanical ventilation.
Sellares et al. (83)	Prospective cohort study (*N* = 181)	To evaluate predictors of prolonged weaning and survival.	Hypercapnia: PaCO_2_ > 45 mmHg	Hypercapnia during the spontaneous breathing trial (SBT), particularly PaCO_2_ ≥ 54 mmHg, was associated with prolonged mechanical ventilation and extubation failure

AHF, acute heart failure; CPD/CPE, cardiogenic pulmonary edema; NIV, non-invasive ventilation; COPD, chronic obstructive pulmonary disease; OHSS, hypopnea–obstructive sleep apnea syndrome; HFNC, high-flow nasal cannula; ARDS, acute respiratory distress syndrome; ALI, acute lung injury; BiPAP, bilevel positive airway pressure; BMI, body mass index; ICU, intensive care unit; IMV, invasive mechanical ventilation; SBT, spontaneous breathing trial; HR, hazard ratio.

Outcomes most frequently reported were mortality (ICU, in-hospital, 30-day, or long-term), NIV failure, re-intubation, duration of mechanical ventilation, and ICU/hospital length of stay. Overall, hypocapnia was commonly associated with worse outcomes in several cohorts, whereas the prognostic signal of hypercapnia appeared more context-dependent, ranging from neutral associations to higher mortality in specific phenotypes such as hypercapnic acidosis early during invasive ventilation.

## 4 Conceptual framework

### 4.1 PaCO_2_ in hypoxemic respiratory failure

In patients with hypoxemic respiratory failure (i.e., PaCO_2_ < 45 mmHg), the most recent guidelines and the latest meta-analysis converge in recommending early HFNC over conventional oxygen therapy and NIV (11–13).

In acute pulmonary edema (APE), recent evidence also supports the use of HFNC (14, 15). However, current guidelines continue to recommend positive-pressure non-invasive support as a first-line strategy, particularly CPAP and, in selected cases, NIV with pressure support (16–18). In this setting, PaCO_2_ appears to function primarily as a physiological and prognostic marker rather than as a direct treatment target. Among patients with acute heart failure and APE, PaCO_2_ < 31 mmHg has been associated with higher mortality, and progressively lower PaCO_2_ levels have been linked to worse outcomes (19). In addition, hypocapnia has been identified as a risk factor for failure of non-invasive respiratory support in patients with cardiogenic pulmonary edema (20) (Figure 1). These findings suggest that low PaCO_2_ may reflect greater respiratory distress and disease severity, rather than representing a variable that should be normalized per se (20). Accordingly, in this context, PaCO_2_ may be useful for risk stratification and clinical assessment, while the therapeutic benefits of positive-pressure support in APE are more directly related to improvements in oxygenation, preload/afterload, and alveolar recruitment than to correction of PaCO_2_ itself (16, 21).

Beyond specific etiologies, PaCO_2_ < 32 mmHg has also been associated with failure of NIV in hypoxemic respiratory failure, and each increment in PaCO_2_ above this threshold has been associated with a lower failure rate in observational analyses (22). In this setting, low PaCO_2_ may reflect increased minute ventilation and heightened respiratory drive (8). Although these physiological features have been linked to excessive inspiratory effort and have been proposed as contributors to patient self-inflicted lung injury, the available evidence in this context is primarily observational and does not establish PaCO_2_ itself as a direct therapeutic target (23–25). Therefore, in predominantly hypoxemic respiratory failure, PaCO_2_ may be better interpreted as an informative physiological and prognostic marker that can support monitoring and risk assessment.

### 4.2 PaCO_2_ in hypercapnic respiratory failure

In patients with hypercapnic acute respiratory failure, particularly when elevated PaCO_2_ is accompanied by respiratory acidosis, the evidence supports non-invasive ventilation as a first-line intervention (18, 26) (Figure 1).

Among patients with acute pulmonary edema (APE), hypercapnia is present in approximately 20–50% (17, 19, 20). In this subgroup, NIV has been associated with a lower need for intubation and reduced in-hospital mortality (20), with reported success rates close to 100% in some series (27–29). Importantly, patients with more severe hypercapnia (PaCO_2_ > 60 mmHg) often require longer durations of NIV, without a corresponding increase in intubation risk (30, 31).

Across etiologies, hypercapnia accompanied by severe acidosis (pH < 7.25) may still justify NIV and can be managed successfully (31). The benefit of NIV has also been demonstrated in hypercapnic encephalopathy (32–34). Similarly, in acute hypercapnic respiratory failure related to obesity and alveolar hypoventilation, NIV has shown clinical benefit, and PaCO_2_ serves as a useful marker to support decision-making, independent of the underlying cause (35–43).

Although studies have suggested potential benefits of high-flow nasal cannula (HFNC) in hypercapnic respiratory failure, lower pH (pH < 7.35 or <7.30) and higher PaCO_2_ (PaCO_2_ > 50 or >59 mmHg) consistently appear to increase the risk of HFNC failure and/or identify patients more likely to benefit from NIV (44–47) (Figure 1). After NIV is initiated, a decrease in PaCO_2_ is a favorable marker of success and may support the start of NIV withdrawal, whereas a rise—or lack of improvement—in PaCO_2_ within the first 4 hours has been proposed as an early predictor of failure and a signal to consider intubation (48–52) (Figure 1).

Taken together, in hypercapnic respiratory failure, PaCO_2_ is identifiable, measurable, and treatable, and it is clinically relevant because it is associated with specific outcomes.

### 4.3 PaCO_2_ in the mechanically ventilated patient

**Figure 2:**
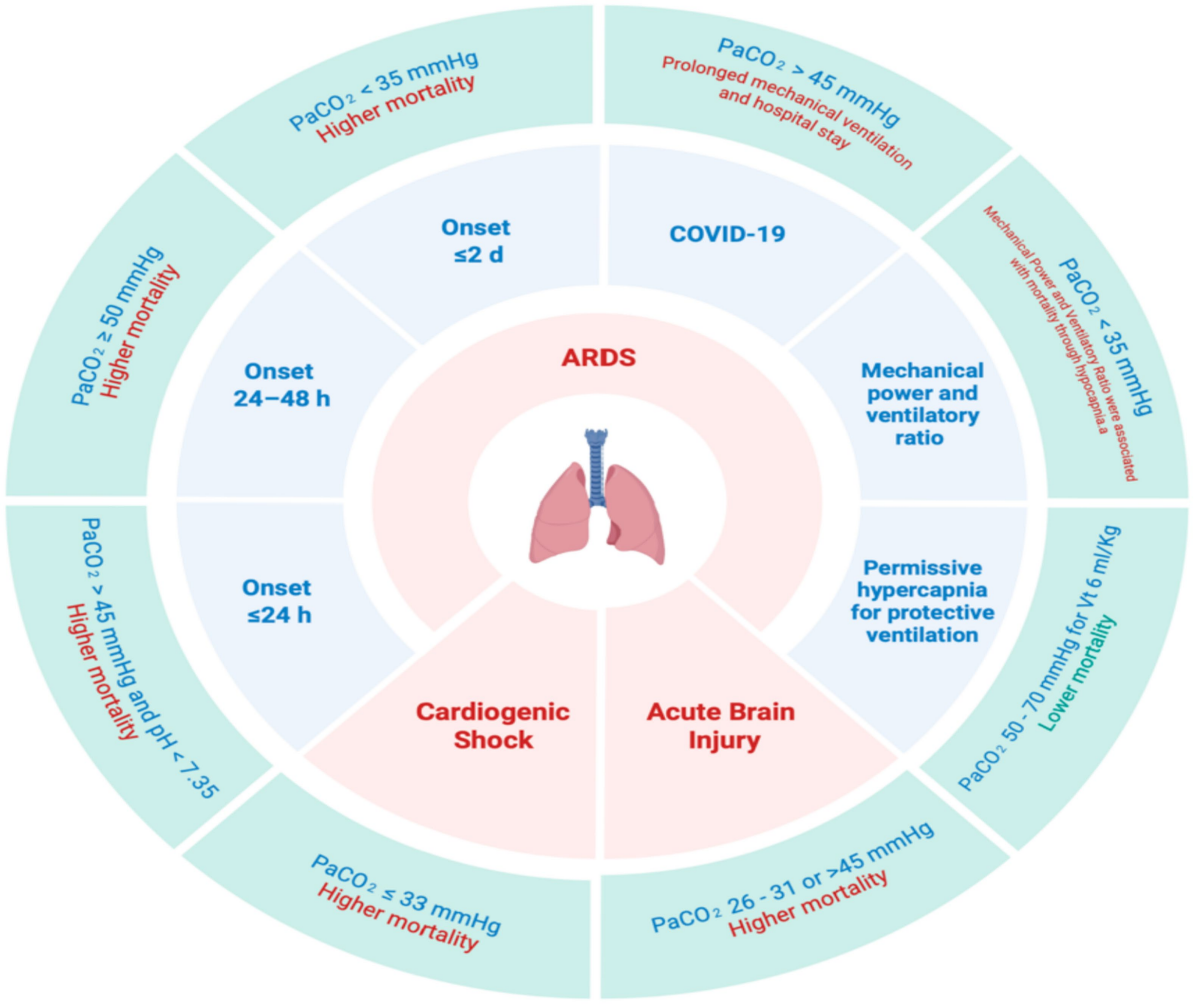
PaCO_2_ as a treatable trait during invasive mechanical ventilation. PaCO_2_ abnormalities during invasive mechanical ventilation are associated with clinically relevant outcomes, but these relationships are highly context-dependent and influenced by the severity of lung injury, ventilatory strategy, and acid–base status. Created by the authors.

Once a patient is intubated, PaCO_2_ remains central to both management and prognosis. However, the clinical significance of a given PaCO_2_ value depends on the underlying context, including the severity of lung injury, ventilatory strategy, and accompanying acid–base status. Observational studies have reported that hypercapnic acidosis early during invasive ventilation is associated with higher mortality, whereas compensated hypercapnia may show a different risk profile (53, 54). These findings are clinically relevant but should be interpreted cautiously, as they may partly reflect confounding by indication in patients with more severe lung injury who require lower tidal volumes and reduced alveolar ventilation. In mechanically ventilated patients with COVID-19, hypercapnia has likewise been associated with longer ventilation duration and longer ICU and hospital length of stay (55), although causality cannot be inferred from these associations alone (Figure 2).

Importantly, the clinical impact of elevated PaCO_2_ cannot be separated from the ventilatory strategy that produces it. A recent meta-analysis suggested that permissive hypercapnia occurring in the context of lung-protective ventilation may be associated with improved mortality, whereas imposed hypercapnia was not, underscoring that the apparent effect of PaCO_2_ is strongly conditioned by the mechanism through which it arises (56). Accordingly, PaCO_2_ should be interpreted less as an isolated determinant of outcome than as a physiological variable embedded within a broader ventilatory and clinical context.

Beyond PaCO_2_ alone, mechanical power and ventilatory ratio are monitoring variables associated with mortality during mechanical ventilation (57–61). Notably, in a cohort of 435 patients with moderate-to-severe ARDS, hypocapnia (PaCO_2_ ≤ 35 mmHg) fully mediated the association between mechanical power and ventilatory ratio with mortality (62) (Figure 2). Consistently, the LUNG SAFE study reported no clear evidence of benefit or harm from hypercapnia, whereas ICU mortality was higher with sustained hypocapnia in mild-to-moderate ARDS (63). Likewise, in mechanically ventilated patients with cardiogenic shock, hypocapnia (PaCO_2_ ≤ 33 mmHg) was associated with an increased risk of 30-day all-cause mortality (64).

In neurocritical care, PaCO_2_ has important pathophysiological effects because it is a major determinant of cerebral vascular tone (65, 66). Decreases in PaCO_2_ promote cerebral vasoconstriction, which may reduce cerebral blood flow and intracranial pressure, whereas increases in PaCO_2_ promote cerebral vasodilation, potentially increasing cerebral blood flow, cerebral blood volume, and intracranial pressure (65, 66). Accordingly, PaCO_2_ abnormalities in patients with acute brain injury may have clinically relevant consequences beyond systemic gas exchange alone (67). In neurocritical care, a “safe” degree of hypocapnia has not been established for patients with traumatic brain injury (TBI). Current guidance recommends against prophylactic hyperventilation to PaCO_2_ < 25 mmHg (68), and Brain Trauma Foundation guidance advises against routine reductions of PaCO_2_ below 30 mmHg (69, 70). In a secondary analysis of a multicenter observational study of 1,476 mechanically ventilated patients with TBI, severe hypocapnia (PaCO_2_ 26–31 mmHg) and hypercapnia (PaCO_2_ > 45 mmHg) were associated with a higher likelihood of in-hospital mortality, whereas normocapnia (PaCO_2_ 35–45 mmHg) and mild hypocapnia (PaCO_2_ 32–35 mmHg) were not associated with worse outcomes (71). Overall, extreme PaCO_2_ values were associated with increased in-hospital mortality in this population (Figure 2).

Prone positioning is among the ARDS interventions with the strongest evidence and recommendations (11, 72). In a retrospective analysis of 225 patients managed with prone positioning, Gattinoni et al. reported that prone responders—defined as those whose PaCO_2_ decreased by ≥1 mmHg after 6 h prone—had higher 28-day survival, suggesting that improved alveolar ventilation (i.e., reduced physiological dead space) may identify patients more likely to survive (73). Several subsequent studies suggested that prone responsiveness may be better captured by PaCO_2_-based metrics than by oxygenation variables (74, 75). More recently, changes in bed positioning have been proposed to improve physiology, ventilatory mechanics, and/or clinical outcomes, and response to these maneuvers has been predicted or demonstrated by changes in PaCO_2_ (76–79).

**Figure 3:**
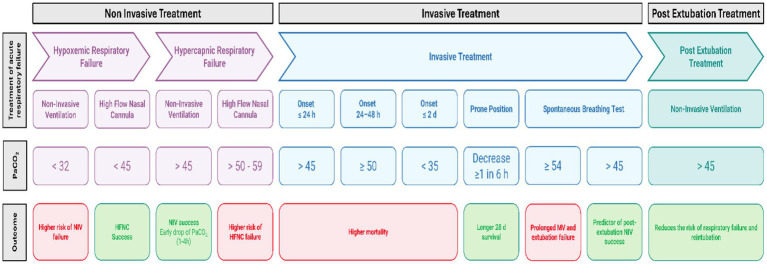
PaCO_2_ timeline as a treatable feature in acute respiratory failure. Created by the authors.

One of the most challenging ICU decisions is liberation from mechanical ventilation, particularly given the risks associated with reintubation. Post-extubation respiratory support remains debated, and to date no clear superiority has been established between HFNC and NIV (80). Therefore, identifying patients at higher risk of failure—and subgroups in whom a specific strategy may prevent reintubation—is clinically important. A systematic review reported that hypercapnia during the spontaneous breathing trial (SBT) was indicative of prolonged weaning or failure (81). In addition, two studies found that hypercapnia during the first SBT, and specifically PaCO_2_ ≥ 54 mmHg during the SBT, were associated with prolonged mechanical ventilation and extubation failure (82, 83). Conversely, hypercapnia (PaCO_2_ > 45 mmHg) during the SBT has also been reported as a predictor of post-extubation NIV success and lower ICU and 90-day mortality (84–86) (Figure 3).

After extubation, PaCO_2_ again becomes a key variable informing the choice of respiratory support. NIV has been shown to be effective in patients with PaCO_2_ > 45 mmHg for preventing post-extubation respiratory failure and reintubation (87, 88). In severe hypercapnia with chronic lung disease, NIV may shorten intubation duration, reduce the risk of post-extubation respiratory failure, and/or prevent reintubation (84, 89, 90) (Figure 3).

Finally, building on the pioneering work of Gattinoni et al. (91, 92), extracorporeal CO_2_ removal has been explored as a strategy to facilitate lower-intensity ventilation and control severe hypercapnia. However, current evidence does not demonstrate a mortality benefit in ARDS. In the REST randomized clinical trial, 90-day mortality was 41.5% in the ECCO_2_R-facilitated lower tidal volume group versus 39.5% in the standard-care group, with no significant difference between groups (93). More recent expert consensus and reviews likewise conclude that ECCO_2_R should not currently be recommended routinely for ARDS outside clinical trials, despite its ability to reduce PaCO_2_ and some ventilatory parameters in physiological studies (94). Thus, in ARDS, ECCO_2_R remains an investigational approach rather than an evidence-based therapy for improving patient-centered outcomes.

In summary, hypocapnia appears deleterious in most mechanically ventilated patients and is associated with increased mortality. Elevated PaCO_2_ may be beneficial in selected patients when it occurs alongside lung-protective ventilation. Pre- and post-extubation PaCO_2_ may inform NIV use and help predict success. Overall, during invasive mechanical ventilation, PaCO_2_ is identifiable and measurable, and it is associated with clinically relevant outcomes; however, its interpretation and potential therapeutic implications remain strongly dependent on the underlying clinical and ventilatory context.

## 5 Conclusion

PaCO_2_ is used to classify respiratory failure, guide non-invasive management, and inform prognosis and risk of treatment failure. Likewise, in intubated patients, PaCO_2_ enables monitoring of ventilatory strategies such as lung-protective ventilation and prone positioning, and it helps contextualize the prognostic value of related variables, including ventilatory ratio and mechanical power. PaCO_2_ also remains clinically relevant before, during, and after liberation from mechanical ventilation, where it can help predict outcomes and support selection of post-extubation non-invasive strategies to reduce the risk of reintubation. Taken together, PaCO_2_ could be considered a treatable trait in critically ill patients with ventilatory failure and warrants further research to clarify its role across the scenarios presented. Future studies should also aim to define safety margins for PaCO_2_ in clinical practice, particularly in relation to pH and exposure time.
